# TET2, ASXL1, IDH1, and IDH2 Single Nucleotide Polymorphisms in Turkish Patients with Chronic Myeloproliferative Neoplasms

**DOI:** 10.4274/tjh.2016.0401

**Published:** 2017-06-01

**Authors:** Nur Soyer, Burçin Tezcanlı Kaymaz, Melda Cömert Özkan, Çağdaş Aktan, Ali Şahin Küçükaslan, Fahri Şahin, Buket Kosova, Güray Saydam

**Affiliations:** 1 Ege University Faculty of Medicine, Department of Hematology, İzmir, Turkey; 2 Ege University Faculty of Medicine, Department of Medical Biology, İzmir, Turkey

**Keywords:** TET2, ASXL1, IDH1, IDH2, Single nucleotide polymorphisms, Ph-negative myeloproliferative neoplasms

## Abstract

We aimed to determine the genotype distribution, allele frequency, and prognostic impact of *IDH1/2, TET2*, and *ASXL1* single nucleotide polymorphisms (SNPs) in myeloproliferative neoplasms (MPNs). *TET2* (rs763480), *ASXL1* (rs2208131), and *IDH1* (rs11554137) variant homozygous genotype frequencies were found at rates of 1.5%, 9.2%, and 2.3%, respectively. No *IDH2* SNP was identified. *IDH1* and *TET2* frequencies were 5% in essential thrombocythemia (ET) and 1.7% in ET and 5% in primary myelofibrosis (PMF), respectively. *ASXL1* frequencies were 8.3%-10% in MPN subgroups. The *TET2* mutant allele T and *ASXL1* mutant allele G had the highest frequencies with 0.272 in the PMF and 0.322 in the polycythemia vera (PV) group, respectively. There was no impact of the SNPs on prognosis. *IDH1* frequency in MPNs was found similar to the literature. *ASXL1* frequencies were similar between ET, PV, and PMF patients. The *ASXL1* and *TET2* allele frequencies of the Turkish population are similar to those of the European population. The role of SNPs in MPNs might be further evaluated in larger multicenter studies.

## Abstract (tr)

Bu çalışmada biz *ASXL1, TET2, IDH1/2* genlerindeki tek nükleotid polimorifizmlerin (SNP) alel sıklığını, genotipik dağılımını ve prognostik etkisini saptamayı amaçladık. *TET2* (rs763480), *ASXL1* (rs2208131) ve *IDH1* (rs11554137) varyant homozigot genotip sıklığı sırasıyla %1,5, %9,2 ve %2,3 saptandı. IDH2 SNP saptanmadı. *IDH1* sıklığı ET’de %5 ve *TET2* sıklığı ET’de %1,7 ve PMF’te %5 idi. *ASXL1* sıklığı ise MPN alt gruplarında %8,3-10’du. En yüksek TET2 mutant allel T ve ASXL mutant allel G sıklığı sırasıyla PMF’te 0,272 ve PV’de 0,322 olarak saptandı. SNP’lerin prognoz üzerine etkisi yoktu. MPN’de IDH1 sıklığı literatür ile uyumlu bulundu. *ASXL1* sıklığı PV, PMF ve ET alt gruplarında benzerdi. Türklerde *ASXL1* ve *TET2* allel sıklığı Avrupalılar ile benzer saptandı. MPN’lerde SNP’lerin rolü, büyük ve çok merkezli çalışmalarda değerlendirilmelidir.

## INTRODUCTION

Philadelphia-negative myeloproliferative neoplasms (MPNs) are clonal disorders classified as polycythemia vera (PV), essential thrombocythemia (ET), and primary myelofibrosis (PMF). MPNs are dependent on hypersensitivity or anomalies in cytokine regulation [1,2]. Some genes have been reported to be involved in the pathogenesis of MPNs, such as *IDH1* (isocitrate dehydrogenase), *IDH2, TET2* (ten-eleven translocation 2), and *ASXL1* (additional sex combs-like 1).

*IDH1/2* encodes enzymes that catalyze the oxidative decarboxylation of isocitrate to α-ketoglutarate [3]. The frequency of *IDH1/2* mutation was 4% in MPNs, 0.8% in ET, 1.9% in PV, and 4.1% in PMF [4,5]. These mutations in PMF were reported as independent predictors of leukemic transformation [6].

*ASXL1* encodes a member of the chromatin-binding proteins and is involved in epigenetic regulation of gene expression [7]. *ASXL1* mutations are rare in ET and PV (<7%) but are frequent in PMF (19-40%) [3,7]. Worsened survival was reported in PMF patients with mutated *ASXL1* [8].

TET proteins are enzymes that can convert 5-methylcytosine to 5-hydroxymethylcytosine [9]. *TET2* mutation frequency was 16% in PV, 5% in ET, and 17% in PMF. Previous research did not identify an impact of *TET2* mutations on survival or leukemic transformation [10].

Although the prognostic impact of these mutations has been investigated in some studies, there is still limited information available [6,8,10]. In this study, we focused only on single nucleotide polymorphisms (SNPs) in the *ASXL1, TET2*, and *IDH1/2* genes. We aimed to determine the genotype distribution, allele frequency, and prognostic impact of selected SNPs in MPNs.

## MATERIALS AND METHODS

The study included 130 MPN patients. The diagnosis of MPNs was performed based on World Health Organization and International Working Group criteria [11]. The Ege University Local Ethics Committee approved the study (13-5.1/8, 15.07.2013). Patient data were collected from the patient files.

SNP analysis was performed on DNA derived from peripheral blood samples that were collected into tubes containing EDTA between February 2008 and September 2009 and were stored at -80 °C until the DNA extraction. DNA extraction was performed using the MagNA Pure Compact Nucleic Acid Isolation Kit (Roche) according to the manufacturer’s instructions. After DNA isolation, the purity and concentration of DNA was measured using a NanoDrop spectrophotometer (Thermo Scientific, USA) at 260 nm and 280 nm.

All SNP analysis was carried out with ready-to-use TaqMan assays from Life Technologies (USA): *IDH1* [rs11554137-(C/T conversion)], *IDH2* [rs121913503-(G/A conversion); rs267606870-(C/G conversion)], *TET2* [rs763480-(A/T conversion)], and *ASXL1* [rs2208131-(A/G conversion). Real-time polymerase chain reaction (PCR) reactions were performed according to the manufacturer’s instructions using the ABI 7500 Fast Real-Time PCR instrument (Applied Biosystems, USA). The real-time PCR cycling conditions were: 95 °C for 10 min for enzyme activation, 40 cycles of 95 °C for 15 s (denaturation), and 60 °C for 1 min (annealing/extension). All assays were evaluated and genotyped using SDS software.

### Statistical Analysis

Hardy-Weinberg equilibrium was used for each SNP. All p-values were two sided and p≤0.05 indicated statistical significance. Categorical and continuous variables were compared with chi-square statistics and the Mann-Whitney U test, respectively. Survival analysis was performed by the Kaplan-Meier method, taking the interval from the date of diagnosis to death or last contact. The log-rank test was used to compare the survival data.

## RESULTS

The demographic features of patients at the time of diagnosis are shown in Table 1. *IDH2* (rs121913503) and *IDH2* (rs267606870) SNPs were not detected in any of the patient groups; all of the cases were genotyped as wild-type homozygous (GG and CC, respectively, for the rs numbers). Two (1.5%) of all, 12 (9.2%) of all, and 3 (2.3%) of all patients were variant homozygous for *TET2* (rs763480), *ASXL1* (rs2208131), and *IDH1* (rs11554137) SNPs, respectively (Table 2).

The *TET2* mutant allele T frequency was 0.218 in the PV, 0.20 in the ET, and 0.272 in the PMF group. The *ASXL1* mutant allele G frequency was 0.322 in the PV, 0.308 in the ET, and 0.25 in the PMF group. The *IDH1* mutant allele T frequency was 0.073 in the PV, 0.108 in the ET, and 0.045 in the PMF group.

**Figure 1:**
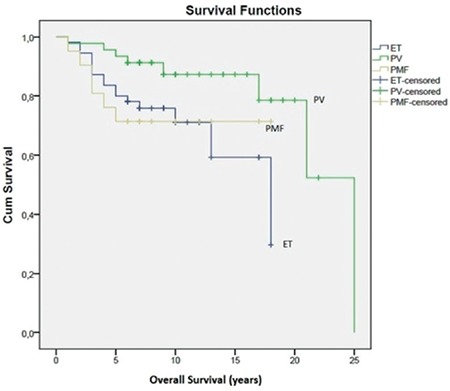
Overall survival of different myeloproliferative neoplasm subgroups. ET: Essential thrombocythemia, PV: polycythemia vera, PMF: primary myelofibrosis.

The median follow-up time was 8 years (range: 1-25). The estimated 10-year survival rate was 71% for ET, 87.3% for PV, and 71% for PMF patients (Figure 1). We did not find any significant differences between the diagnostic subgroups and the 10-year survival rates. At the time of the analysis, 5 and 10 patients had leukemic transformation and fibrotic transformation, respectively. There were no significant differences between the sexes, diagnoses, *JAK2* mutation status (positive/negative), thrombosis status at diagnosis, survival rates, leukemic and fibrotic transformation, and genotyping results of *ASXL1, TET2*, and *IDH1* (Table 3).

## DISCUSSION

In this study, we aimed to determine the genotype distribution, allele frequency, and prognostic impact of selected SNPs in MPNs. IDH mutation frequency was 2.5% in MPNs, 0.8% in ET, 1.9% in PV, 4.1% in PMF, and 1% in post-ET/PV-myelofibrosis patients [5]. Another study reported 3.70% *IDH1* (G105G allele), 1.85% *IDH2* R140Q, and 0.92% *IDH2* (G145G allele) mutation in MPNs [12]. The frequency of the *IDH1* (rs11554137) SNP in our cases was similar to that in the literature.

*TET2* mutation frequency was 7.2%-13% in MPNs and was similar across different MPN subgroups. *JAK2*-positive patients (17%) had significantly higher *TET2* mutation frequency [10,13]. In our series, *TET2* (rs763480) frequency was higher in PMF patients.

*ASXL1* mutation frequency was 10% in MPN patients. These mutations are rare in ET and PV (<7%) but frequent in PMF (19%-40%) [3,7]. In *JAK2*- and *MPL*-negative MPN patients, *TET2* and *ASXL1* mutation frequencies were 8% [14]. *ASXL1* mutation frequency was 24.7% in PMF and 8.4% in ET patients [15]. *ASXL1* mutation frequency was 12%-13% in PMF patients [16]. The frequency of the *ASXL1* (rs2208131) SNP was 9.2% and this was similar between ET, PV, and PMF patients. We did not find any previous study that evaluated *ASXL1* (rs2208131) and *TET2* (rs763480) SNPs in MPNs.

There was no relationship between *TET2, IDH1*, and *ASXL1* SNPs and clinical and laboratory factors in our study. An impact of *TET2* mutation on survival and leukemic transformation was not shown [10]. In normal karyotype acute myeloid leukemia, the *IDH1* (rs11554137) SNP was an adverse prognostic factor [17]. In PMF, there was a significantly negative impact of *IDH* mutations on survival [6]. *ASXL1* mutations were identified in patients with PMF or post-ET/PV-myelofibrosis and associated with poor survival [8,9,15]. Whether the *TET2, IDH1*, and *ASXL1* SNPs possibly confer any prognostic impact in MPN patients requires further evaluation.

The *TET2* mutant allele T and *ASXL1* mutant allele G had the highest frequencies at 0.272 in the PMF and 0.322 in the PV group, respectively. The *ASXL1* and *TET2* mutational statuses in the Turkish population are similar to those of the European population according to HapMap CEU data reporting 0.322 G mutant allele and 0.372 T mutant allele frequencies, respectively (hapmap.ncbi.nlm.nih.gov/).

One of the limitations of this study is the small sample size. Since we did not evaluate *MPL* and *CALR* mutations, we could not assess whether there was a relationship between *CALR* and *MPL* mutations and these SNPs. Since we had no control group, we could not perform a comparison for these SNPs between patients and controls.

## CONCLUSION

*IDH1* frequency in MPNs was found to be similar to the rate reported in the literature. *ASXL1* frequencies were similar between ET, PV, and PMF patients. We did not find an impact of the SNPs on survival, *JAK2* status, or leukemic and fibrotic transformation. These findings suggest that *IDH1* is a rare SNP in MPNs. The role of SNPs in MPNs might be further evaluated in larger multicenter studies.

## Figures and Tables

**Table 1:**
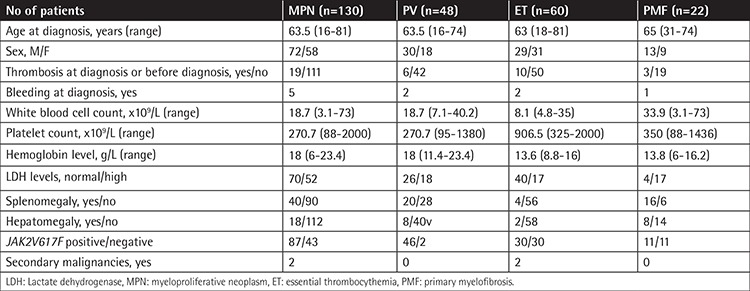
The demographic features of myeloproliferative neoplasm patients at the time of diagnosis.

**Table 2:**
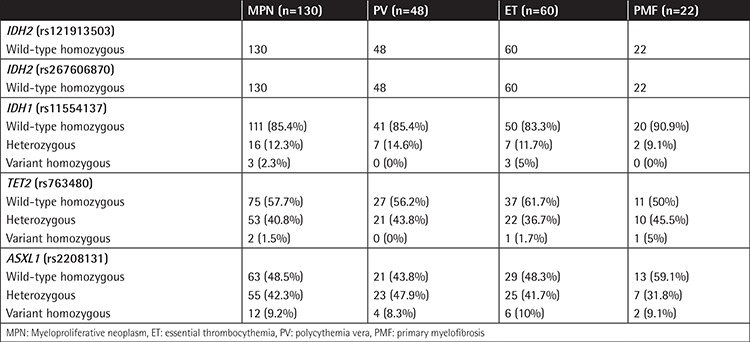
Genotype status of *IDH1, IDH2, TET2*, and *ASXL1* single nucleotide polymorphisms in myeloproliferative neoplasm and myeloproliferative neoplasm subgroups.

**Table 3:**
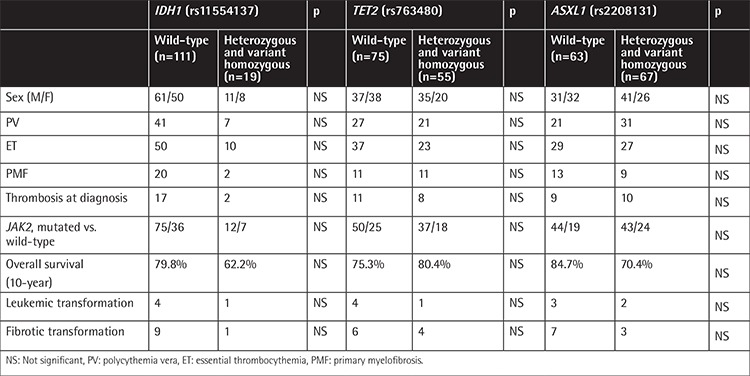
Genotype status of *IDH1, TET2*, and *ASXL1* single nucleotide polymorphisms and clinical and laboratory correlations.
